# Comparison of anakinra and tocilizumab in management of severe COVID-19: a retrospective cohort study

**DOI:** 10.55730/1300-0144.5487

**Published:** 2022-06-26

**Authors:** Orhan KÜÇÜKŞAHİN, Abdulsamet ERDEN, Özlem KARAKAŞ, Serdar Can GÜVEN, Berkan ARMAĞAN, Enes Seyda ŞAHİNER, Osman İNAN, Ali Can KURTİPEK, Emin GEMCİOĞLU, Selma KARAAHMETOĞLU, Sema TURAN, Seval İZDEŞ, Deniz ERDEM, Adalet AYPAK, Müge AYHAN, Esragül AKINCI, Hürrem BODUR, Rahmet GÜNER, Ahmet OMMA, İhsan ATEŞ

**Affiliations:** 1 Department of Internal Medicine, Division of Rheumatology, Faculty of Medicine, Ankara Yıldırım Beyazıt University, Ankara, Turkey; 2 Clinic of Rheumatology, Ministry of Health Ankara City Hospital, Ankara, Turkey; 3 Clinic of Internal Medicine, Ministry of Health Ankara City Hospital, Ankara, Turkey; 4 Clinic of Intensive Care Unit, Ministry of Health Ankara City Hospital, Ankara, Turkey; 5 Department of Anesthesiology and Reanimation-Critical Care, Faculty of Medicine, Ankara Yıldırım Beyazıt University, Ankara, Turkey; 6 Clinic of Anesthesia and Resuscitation, Ministry of Health Ankara City Hospital, Ankara, Turkey; 7 Clinic of Infectious Diseases and Clinical Microbiology, Ministry of Health Ankara City Hospital, Ankara, Turkey; 8 Department of Infectious Diseases and Clinical Microbiology, Faculty of Medicine, Health Sciences University, Ankara City Hospital, Ankara, Turkey; 9 Department of Infectious Diseases and Clinical Microbiology, Faculty of Medicine, Ankara Yıldırım Beyazıt University, Ankara, Turkey; 10 Department of Internal Medicine, Division of Rheumatology, Faculty of Medicine, Health Sciences University, Ankara City Hospital, Ankara, Turkey; 11 Department of Internal Medicine, Faculty of Medicine, Health Sciences University, Ankara City Hospital, Ankara, Turkey

**Keywords:** COVID-19, cytokine storm, hyperinflammation, anakinra, tocilizumab

## Abstract

**Background/aim:**

Studies regarding effectiveness of anakinra and tocilizumab treatments in coronavirus disease 2019 (COVID-19) have contradictory results. Furthermore, there is scarce comparative data regarding superiority of any agent. To further elucidate any superiority between these two agents, we retrospectively investigated and compared outcomes in hospitalized COVID-19 patients of our inpatient cohort who received anakinra or tocilizumab.

**Materials and methods:**

This study was designed as a single-center, retrospective, cross-sectional cohort study. Hospitalized patients with confirmed diagnosis of COVID-19 who had Brescia-COVID respiratory severity scale score ≥3 and hyperinflammation (defined as elevation of C reactive protein ≥50 g/L or ferritin ≥700 ng/mL) and received anakinra or tocilizumab in addition to standard care were enrolled in the study. Length of hospital stay after initiation of antiinflammatory treatment, need for mechanical ventilation, need for intensive care unit admission, mortality were set as primary outcomes and compared between tocilizumab and anakinra recipients after propensity score matching.

**Results:**

One hundred and six patients were placed in each group after propensity score matching. In the anakinra group, relative risk reduction for intensive care unit admission was 50% when compared to the tocilizumab group and the number needed to treat to avert an intensive care unit admission was 3 (95% CI, 2–5). In terms of mortality, a 52% relative risk reduction was observed with anakinra treatment and the number needed to treat to avert an intensive care unit admission was 8 (95% CI, 4–50). Significantly more patients were observed to receive glucocorticoids in the anakinra group.

**Conclusion:**

Anakinra administration in severe COVID-19 patients was significantly associated with better survival and greater clinical improvement compared to the tocilizumab administration in our study. Increased rate of glucocorticoid use in the anakinra group might have contributed to better outcomes.

## 1. Introduction

Coronavirus disease 2019 (COVID-19) has a wide spectrum of clinical presentations with disease course varying from asymptomatic infection to severe pneumonia causing death [1]. Approximately 5% of COVID-19 cases need intensive care due to systemic inflammation and acute respiratory distress syndrome (ARDS) with a mortality rate of 1%–2% [2]. Proinflammatory cytokines such as interferon (IFN) γ, tumor necrosis factor (TNF)-α, interleukin (IL)-6, IL-1β, and IL-18 play a major role in immune response to viral infections; however, triggering of a hyperimmune state characterized by excessive and dysregulated release of these cytokines, so called “cytokine storm”, has been defined as a major cause of mortality and morbidity in COVID-19.

Cytokine storm has been revealed to correlate with disease severity in hospitalized COVID-19 patients and defined as a prognostic factor for worse outcomes [3–5]. Therefore, undelayed recognition of cytokine storm and administration of appropriate antiinflammatory agents with optimal timing seems to be crucial. Considerable knowledge regarding antiinflammatory treatment strategies such as systemic corticosteroids, colchicine, anticytokine agents, and intravenous immunoglobulins has been accumulated in the literature during the pandemic; however, precise approach for management of cytokine storm is yet to be fully clarified.

Interleukin-1 and IL-6 are proinflammatory cytokines which have been demonstrated to be major contributors to development of hyperinflammatory response in COVID-19. Interleukin-1a is one of the initial cytokines released in COVID-19, inducing IL-6, TNF-α, granulocyte-macrophage colony stimulating factor (GM-CSF), and IL-17 expression after binding to its receptor [6]. Likewise, overproduction of IL-6 reported to drive the immune dysregulation in severe COVID-19 patients [7]. Both these cytokines seem to be potential targets for management of cytokine storm in COVID-19. Accordingly, blockers of both IL-1 and IL-6 have been investigated in treatment of COVID-19.

Tocilizumab is a recombinant humanized monoclonal antibody directed against soluble and membrane-bound IL-6 receptors, and anakinra is a recombinant human IL-1 receptor antagonist. Effectiveness of both tocilizumab and anakinra on outcomes has been reported alongside contradictory results; nevertheless, both agents have been used in management of COVID-19 during the pandemic [8–19]. In addition to the controversy surrounding the effectiveness of these agents, there is scarce comparative data regarding superiority of any anticytokine agent in COVID-19 [20].

To further elucidate any superiority between these two agents, here in this study, we retrospectively investigated and compared outcomes in hospitalized COVID-19 patients of our inpatient cohort who received anakinra or tocilizumab treatments.

## 2. Materials and methods

This is a single-center, retrospective, cross-sectional study. Ethical approval of the study was obtained from the Ethics Committee of Ankara City Hospital. Hospitalized adult COVID-19 patients between April 1 and December 31, 2020, from Ankara City Hospital Internal Medicine, Infectious Diseases and Clinical Microbiology, Critical Care clinics were retrospectively investigated. Among these, patients with confirmed diagnosis of COVID-19 (positive severe acute respiratory syndrome coronavirus 2 (SARS-CoV-2) polymerase chain reaction test with compatible findings in chest X-ray or computed tomography) who had Brescia-COVID respiratory severity scale (BCRSS) score ≥3 and hyperinflammation (defined as elevation of C-reactive protein (CRP) ≥50 mg/L or ferritin ≥700 ng/mL) and received anakinra or tocilizumab treatments in addition to standard care were enrolled in the study [21]. Patients who were under the age of 18, pregnant, or had a history of biologic agent or systemic glucocorticoid use prior to COVID-19, a malignant disease, or a psychiatric disorder at the time of hospitalization were excluded.

In our center during the pandemic, hospitalization, treatment, management, and discharge decisions of the patients were made according to the national guidelines prepared by Turkish Health Ministry^1^. In accordance with these guidelines, standard care in our center comprised hydroxychloroquine (400 mg/day without loading for 5 days and continued for 10 days in severe cases), low molecular weight heparin, acetylsalicylic acid, favipiravir (3200 mg on day 1 and 1200 mg on days 2–5). All patients in this study were managed in guidance with these regulations and received standard care medications if not contraindicated. Likewise, administration of antiinflammatory treatment agents including systemic glucocorticoids, tocilizumab, anakinra, and intravenous immunoglobulins were decided according to national guidelines. Anakinra dose varied from 2 to 10 mg/kg and administered subcutaneously in divided doses for every 6 h [16]. Duration (days) of anakinra treatment was decided by attending resident and individualized according to response and side effects. Tocilizumab was administered a single dose of 8 mg/kg via peripheral intravenous route [11,12]. After initial administration, a second dose was administered after 24/48 h in refractory patients if deemed necessary by attending resident [12,22].

Demographic, clinical, laboratory, imaging, treatment, and outcome data were collected using a standardized form. All data were saved by the same physician (OK). Patients were grouped into two as anakinra recipients and tocilizumab recipients. Length of hospital stay after initiation of antiinflammatory treatment, need for mechanical ventilation, need for intensive care unit (ICU) admission, and mortality were set as primary outcomes and compared between groups after propensity score (PPS) matching.

Data were analyzed using SPSS v. 22.0 software except for propensity score matching which was done with the statistical software R (version 3.5.1, The R Foundation for statistical computing, Vienna, Austria). Shapiro–Wilk’s test was used to determine the distribution of the data. The distribution of continuous data was expressed as mean ± standard deviation. Continuous variables that did not conform to normal distribution were expressed as median and interquartile range (IQR) values. Continuous variable was compared by using either Student’s t-test or the Mann–Whitney U test according to normality. For categorical variables, χ^2^ test was used and the outcomes were expressed as number and percentages. Relative risk (RR) values and their 95% confidence interval (CI) were calculated through crosstabs. p-values below 0.05 were considered statistically significant. We calculated the propensity between two groups on the basis number of days from symptom onset to administration of anticytokine treatment used in the logistic-regression analysis. We matched each patient in the tocilizumab group to the patient in the anakinra group (1:1) with the closest propensity score, using a greedy nearest neighbor matching without replacement within 0.001 caliper widths.

## 3. Results

A total of 360 patients were enrolled in the study (120 anakinra recipients, 240 tocilizumab recipients). Demographics, comorbidities, COVID-19 symptoms, and days from symptom onset to administration of anticytokine treatment are given in Table 1. Days from symptom onset to administration of anakinra or tocilizumab were significantly different between groups (median (IQR) 10 (6) vs 8 (6) day, p < 0.0001, respectively). After PPS matching based on duration of symptom onset, 106 patients were placed in both groups. No significant differences were observed between groups in means of sex and comorbidities before and after PPS matching (Table 1). Cough (60.4% vs 42.5%, p = 0.013) and dyspnea (75.5% vs 55.7%, p = 0.04) were significantly more common in the tocilizumab group after matching.

Baseline and last laboratory parameters of both groups before and after PPS matching are given in Table 2. Baseline CRP, D-dimer, and lactate dehydrogenase levels remained significantly higher in the tocilizumab group after PPS match. In last parameters, CRP levels were similar between groups, fibrinogen was higher, and D-dimer was significantly lower in the anakinra group after PPS match (Table 2).

Initial median (IQR) anakinra dose was found to be 600 (200) mg and median (IQR) duration of treatment was 7 (4) days before and after PPS matching. Median (IQR) tocilizumab dose for first administration was 400 (200) mg and median (IQR) duration of treatment was 2 (1) days.

Primary outcomes are presented in Table 3. Duration of hospital stay after onset of treatment administration was significantly longer in the anakinra group both before and after PPS matching (median (IQR), 11 (11) days vs 10 (8) days, p = 0.03, after PPS matching). However, length of hospital stay after end of anticytokine treatment was reduced in the anakinra group (median (IQR), 4 (12) days vs 8.5 (9) days, p ≤ 0.0001, after PPS matching). Mechanical ventilation rates were similar between groups. Rate of intensive care unit admission was lower in the anakinra group both before (35% vs 66.7%, p < 0.001) and after (33% vs 66%, p < 0.001) PPS matching. Likewise, mortality levels were lower in the anakinra group before and after PPS matching (14.2% vs 22.9%, p = 0.05 and 11.3% vs 23.6%, p = 0.029, respectively).

Rates of favipiravir, hydroxychloroquine, and glucocorticoids are presented in Table 4. After PPS, frequency of hydroxychloroquine use was similar between groups and all patients were observed to receive favipiravir. Rate of glucocorticoid use was significantly higher in the anakinra group (98.1% vs 79.2%, p < 0.0001).

After propensity score matching, in the intention-to-treat (ITT) population, intensive care unit admission developed in 35 of 106 patients (33%) who received anakinra and in 70 of 106 patients (66%) who received tocilizumab (relative risk, 0.50; 95% CI, 0.36 to 0.67; p< 0.001). The relative risk reduction with anakinra was 50% and the number needed to treat (NNT) to avert an intensive care unit admission was found to be 3 (95% CI, 2 to 5). After propensity score matching, in the ITT population, mortality developed in 12 of 106 patients (11.3%) who received anakinra and in 25 of 106 patients (23.6%) who received tocilizumab (relative risk, 0.48; 95% CI, 0.25 to 0.90; p = 0.029). The relative risk reduction with anakinra was 52% and the NNT to avert a mortality was found to be 8 (95% CI, 4 to 50).

## 4. Discussion

In this retrospective cohort study, we evaluated the outcomes of tocilizumab and anakinra treatments in severe (BCRSS ≥ 3) COVID-19 patients. Both tocilizumab and anakinra groups were similar in terms of age, sex, and comorbidity. After PPS matching based on days from symptom onset to initiation of anticytokine treatment, rates of mortality and admission to ICU were significantly lower in the anakinra group. Rate of additional glucocorticoid use was significantly higher in the anakinra group.

COVID-19 generally causes mild illness; however, a substantial number of patients suffer from severe respiratory distress leading to death [23]. SARS-CoV-2 induces a cytokine storm similar to macrophage activation syndrome characterized by excessive expression of proinflammatory cytokines [24]. Viral proteins induce inflammasome and caspase-1 activation via binding to toll-like receptors. Caspase 1 cleaves inactive pro-IL-1β to active IL-1β which is an important mediator for lung inflammation and fibrosis in COVID-19 [25]. Furthermore, IL-1β mediates Il-6 synthesis, a key proinflammatory cytokine in COVID-19 cytokine storm and a potent inducer of CRP [26]. Virus-infected monocytes, macrophages, and dendritic cells further induce expression of IL-6 and other proinflammatory cytokines. Presence of such hyperinflammatory state is accepted as an indicator for a COVID-19 infection [27]. Growing evidence suggest that severe COVID-19 patients have higher plasma levels of cytokines, such as IL-1β, IL-6, and IL-10, and concentration of these cytokines in the plasma may differentiate mild, moderate, and severe cases [28,29]. Cytokine storm has been associated with development of ARDS, disseminated intravascular coagulation, and multiorgan failure [27].

Immune dysregulation in COVID-19 is reported to be mediated by IL-6 and in animal models low IL-6 concentrations were associated with less severe acute lung injury [30]. Interleukin-6 may be a potential target in treatment of COVID-19–related cytokine storm. Accordingly, tocilizumab treatment reported to reduce the need for mechanical ventilation, improve hypoxemia, and regress imaging findings [13]. Similarly, Toniati et al. [14] related tocilizumab treatment with clinic improvement in ¾ of 100 COVID-19 patients. In a retrospective cohort of 544 patients, Guaraldi et al. [15] reported a significantly lower death rate in the tocilizumab group compared to the control group (7% vs 20%, respectively). Among 764 COVID-19 patients in the ICU, Biran et al. [31] reported a significant decrease in the mortality rate and risk of mechanical ventilation with tocilizumab. In contrast to these observational findings, in five randomized controlled trials, no superiority against placebo or standard care with tocilizumab was observed except for EMPACTA study which revealed benefit of tocilizumab in reducing the likelihood of progression to requiring mechanical ventilation or death [8–12]. In addition, a systematic review and metaanalysis of seven retrospective studies showed that there is no statistically significant difference between tocilizumab and standard care in terms of all-cause mortality (odds ratio (OR): 0.62; 95% CI: 0.31–1.22) and ICU admission (relative risk (RR): 1.51; 95% CI: 0.33–6.78) [32].

Interleukin-1 also seems to be an important cytokine in pathogenesis of COVID-19–related cytokine storm. Increased IL-1α and IL-1β expression was revealed in severe COVID-19 patients prior to development of respiratory distress [24]. Furthermore, COVID-19–related cytokine storm shares similarities with other autoinflammatory conditions characterized by hyperinflammation such as adult onset Still’s disease and macrophage activation syndrome in which IL-1 pathway is a major target for treatment [33,34]. Therefore, anakinra has been considered for treatment of COVID-19–related cytokine storm and several studies demonstrated beneficial effects [35]. A retrospective study analyzed 29 severe COVID-19 patients treated intravenously with anakinra and the majority of which had pO2/FiO2 less than 100 mmHg [16]. The mortality rate of this study was 10% on the 21st day which was significantly lower compared to the 16 comparator patients with a mortality rate of 44% who received standard-of-care treatment. Huet et al. [17] reported reduced mortality and mechanical ventilation rates in severe COVID-19 pneumonia with a 10-day course of anakinra when compared to nonusers. Likewise, Balkhair et al. [18] related anakinra treatment with reduced mortality and increased rate of successful weaning from mechanical ventilation in severe COVID-19 patients. However, in randomized controlled CORIMUNO-ANA-1 study, no benefit of anakinra was observed in mild and moderate COVID-19 patients on invasive or noninvasive mechanical ventilation support [19].

There is scarce comparative data in the literature regarding superiority of anakinra and tocilizumab to each other in management of COVID-19. In a recently published retrospective study with comparison of IL-1 inhibition (anakinra) and IL-6 inhibition (tocilizumab or sarilumab) strategies, IL-1 inhibition but not IL-6 inhibition was reported to reduce mortality, respiratory insufficiency, and hyperinflammation significantly in hospitalized COVID-19 patients [20]. In our study, we observed better outcomes in means of mortality and ICU admission rates with anakinra. In the anakinra group, relative risk reduction for ICU admission was 50% when compared to tocilizumab group and NNT to avert an ICU admission was 3 (95% CI, 2 to 5). In terms of mortality, a 52% relative risk reduction was observed with anakinra treatment and NNT to avert was 8 (95% CI, 4 to 50). Hospital stay after initiation of anticytokine treatment was shorter in tocilizumab group (10 (8) days vs 11 (11) days, p = 0.003) and we assume this may be related to increased mortality in tocilizumab group. When baseline and last laboratory parameters were evaluated (Table 2), tocilizumab seemed to provide better reduction in inflammatory markers; however, this reduction was not coherent with outcomes since anakinra group had reduced mortality and ICU admission.

In management of autoinflammatory conditions, anakinra was generally administered with a dose of 100 mg/day subcutaneously [36]. Several data suggest higher doses (100–400 mg/day) and intravenous administration in management of life threatening hyperinflammatory conditions such as macrophage activation syndrome, and due to short half-life (3 h), diving daily total dose to 6 h intervals are suggested in severe conditions [37,38]. In our study, median (IQR) anakinra dose at the onset of treatment was 600 (200) mg/day and median (IQR) duration of treatment was 7 (4) days.

The RECOVERY group demonstrated effects of dexamethasone on mortality in COVID-19 and glucocorticoids have been used widespread ever since in treatment of COVID-19 [39]. In our study, in the anakinra group, rate of glucocorticoid use was significantly higher, which may indicate contributory effects of glucocorticoid use and may imply better outcomes with combination of anakinra and glucocorticoids when compared to tocilizumab. This result may be due to the fact that beneficial effects of tocilizumab in COVID-19 were reported earlier than use of anakinra and glucocorticoids.

There are several limitations in our study. Firstly, this was a retrospective observational study, which might aggravate the risk of bias and causal inferences cannot be drawn because of inherent known and unknown confounders. Secondly, there was a possibility of sampling bias since we obtained data from a convenience sample. A major limitation was that the use of glucocorticoids, even after PPS matching, was more frequent in the anakinra group; therefore, it cannot be differentiated whether better outcomes in the anakinra group was a result of anakinra use or anakinra and glucocorticoid combination. Additionally, the frequency of cough, dyspnea, and baseline levels of CRP, fibrinogen, and D-dimer were higher in the tocilizumab group, so we cannot elude the possibility of higher disease severity in the tocilizumab group. Finally, adverse effects were not evaluated for neither of the agents.

In conclusion, in our study, survival was better and clinical improvement was greater in severe COVID-19 patients who received anakinra when compared to those who received tocilizumab. Glucocorticoid use was significantly more frequent in the anakinra group, which may be indicative of better outcomes with anakinra and glucocorticoid combination. Furthermore, there was a chance that disease severity was higher in the tocilizumab recipients. There are several factors which may interfere with effectiveness of anticytokine treatments in COVID-19 such as dosing and timing of initiation. Future studies are needed to optimize anticytokine treatment strategies in COVID-19.

## Figures and Tables

**Table 1:** Demographics, comorbidities, and COVID-19 symptoms in tocilizumab and anakinra recipients.

	All patients			After propensity score			
	Anakinra (n:120)	Tocilizumab (n: 240)	p	Anakinra (n: 106)	Tocilizumab (n: 106)	p	
**Male sex, n (%)**	98 (81.7)	182 (75.8)	0.209	87 (82.1)	83 (78.3)	0.491	
**Median age, years, (IQR)**	57 (15)	59 (16)	0.109	56 (15)	59 (16)	0.127	
**COVID-19 symptoms, n (%)**							
Cough	51 (42.5)	130 (54.2)	0.037	45 (42.5)	64 (60.4)	0.013	
Fever	66 (55)	126 (52.5)	0.654	62 (58.5)	56 (52.8)	0.49	
Dyspnea	70 (58.3)	158 (65.8)	0.164	59 (55.7)	80 (75.5)	0.04	
Headache	12 (10)	15 (6.3)	0.203	11 (10.4)	7 (6.6)	0.46	
Back pain	7 (5.8)	4 (1.7)	0.03	7 (6.6)	1 (0.9)	0.06	
Arthralgia	5 (4.2)	12 (5)	0.725	5 (4.7)	7 (6.6)	0.76	
Myalgia	67 (55.8)	95 (39.6)	0.003	61 (57.5)	43 (40.6)	0.01	
Anosmia	2 (1.7)	15 (6.3)	0.065	2 (1.9)	6 (5.7)	0.28	
Ageusia	1 (0.8)	24 (10)	0.001	1 (0.9)	10 (9.4)	0.01	
**Comorbidities, n (%)**							
Patients with ≥1 comorbidities	77 (64.2)	154 (64.2)	1	66 (62.3)	66 (62.3)	1	
Hypertension	51 (42.5)	104 (43.3)	0.88	43 (40.6)	43 (40.6)	1	
Diabetes	31 (25.8)	73 (30.4)	0.366	26 (24.5)	32 (30.2)	0.44	
Asthma	9 (7.5)	15 (6.3)	0.654	9 (8.5)	7 (6.6)	0.79	
COPD	4 (3.3)	13 (5.4)	0.38	4 (3.8)	7 (6.6)	0.53	
CHD	27 (22.5)	45 (18.8)	0.402	19 (17.9)	20 (18.9)	1	
Renal disease	5 (4.2)	12 (5)	0.725	3 (2.8)	5 (4.7)	0.72	
**Days from symptom onset to treatment administration, median (IQR)**	10 (6)	8 (6)	<0.0001	10 (4)	10 (4)	1	

n: number; IQR: interquartile range; COVID-19: coronavirus disease 2019; COPD: chronic obstructive pulmonary disease; CHD: chronic heart disease

**Table 2:** Baseline and last laboratory parameters in tocilizumab and anakinra recipients.

	Baseline						Last					
	Before propensity score			After propensity score			Before propensity score			After propensity score		
	Anakinra (n:120)	Tocilizumab (n:240)	p	Anakinra (n:106)	Tocilizumab (n:106)	p	Anakinra (n:120)	Tocilizumab (n:240)	p	Anakinra (n:106)	Tocilizumab (n:106)	p
Creatinin, mg/dL	0.88 (0.3)	0.82 (0.3)	0.034	0.89 (0.27)	0.82 (0.28)	0.027	0.87 (0.29)	0.85 (0.35)	0.711	0.88 (0.25)	0.86 (0.33)	0.649
AST, U/L	40 (30)	50 (42)	<0.0001	41 (30)	48 (44)	0.07	34 (28)	37 (52)	0.132	35 (28)	36 (50)	0.289
ALT, U/L	44 (42)	44 (42)	0.329	45 (42)	43 (43)	0.582	64 (56)	64.5 (85)	0.889	66.5 (54)	63.5 (73)	0.779
LDH, U/L	400 (219)	476 (245)	0.001	398 (189)	492 (244)	0.001	301 (145)	317 (276)	0.156	292 (133)	312 (284)	0.270
CRP, mg/L	81 (99)	126 (90.5)	<0.001	83.5 (97.7)	127.5 (87.7)	0.003	8 (25.2)	5 (18)	0.037	7 (21.5)	5 (19)	0.196
ESR, mm/h	35.5 (31.5)	51 (39.75)	<0.001	36 (35.5)	46.5 (46.5)	0.01	24 (32)	17 (35)	0.071	25 (37)	16.5 (34)	0.067
Ferritin, μg/L	718 (829)	950 (931)	0.032	723 (776)	873 (837)	0.188	407 (515)	465 (577)	0.623	405 (473)	429 (469)	0.792
WBC, 10^9^/ L	9.74 (5.06)	8 (4.68)	0.001	9.9 (5.4)	7.59 (3.9)	<0.001	8.61 (4.6)	8.06 (6.1)	0.565	8.45 (4.53)	7.9 (6.78)	0.438
Lymphocyte, 10^9^/ L	0.59 (0.39)	0.76 (0.46)	<0.001	0.59 (0.39)	0.77 (0.45)	0.001	1.51 (1.05)	1.44 (1.03)	0.328	1.53 (0.87)	1.41 (0.99)	0.124
Albumin, g/dL	35.9 (7.52)	35.24 (6)	0.338	36.4 (6.4)	35 (6.5)	0.018	35.8 (10.2)	35 (7)	0.151	37.02 (9.6)	34.2 (7.2	0.036
CK, U/L	78.5 (110.25)	119.5 (213)	<0.0001	79 (119)	101 (132)	0.03	46 (68)	51 (113)	0.163	49 (67)	46 (58)	0.855
D-dimer, mg/L	0.63 (1.41)	1.09 (1.89)	<0.0001	0.59 (1.2)	1.14 (2.15)	<0.001	0.49 (0.87)	0.97 (2.64)	<0.0001	0.46 (0.75)	0.87 (3.35)	<0.0001
Fibrinogen,g/L	5.46 (2.36)	5.68 (2.23)	0.494	5.6 (2.41)	5.6 (2.47)	0.43	3.79 (1.63)	2.69 (1.97)	<0.0001	3.9 (1.62)	2.69 (1.95)	<0.0001
Hg, g/dL	13.4 (1.8)	13.2 (2.2)	0.272	13.35 (1.9)	13.1 (2.4)	0.078	13 (2.9)	13 (2.7)	0.92	13.15 (2.65)	12.9 (2.53)	0.623
Platelet, 10^9^/L	229 (141)	274.5 (165)	0.002	236 (136)	273 (160)	0.02	285 (148)	279 (148)	0.639	286.5 (140)	266 (167)	0.414

AST: aspartate transaminase; ALT: alanine transaminase; LDH: lactate dehydrogenase; CRP: c-reactive protein; ESR: eritrocyte sedimentation rate; WBC: white blood cells; CK: creatine kinase; Hg: hemoglobin

**Table 3:** Outcomes in tocilizumab and anakinra recipients.

	All patients			After propensity score		
	Anakinra (n:120)	Tocilizumab (n:240)	p	Anakinra (n:106)	Tocilizumab (n:106)	p
Length of hospital stay after treatment initiation, days, median (IQR)	12 (12)	11 (9)	0.027	11 (11)	10 (8)	0.03
Length of hospital stay after treatment ends, days, median (IQR)	4 (12)	9 (9)	<0.0001	4 (12)	8.5 (9)	<0.0001
Rate of invasive mechanical ventilation, n(%)	32 (26.7)	68 (28.3)	0.73	25 (23.6)	30 (28.3)	0.53
Rate of intensive care unit admission, n(%)	42 (35)	160 (66.7)	<0.0001	35 (33)	70 (66)	<0.001
Mortality, n(%)	17 (14.2)	55 (22.9)	0.05	12 (11.3)	25 (23.6)	0.029

n:number; IQR: interquartile range

**Table 4:** Frequencies of favipiravir, hydroxychloroquine, and glucocorticoid use in tocilizumab and anakinra recipients.

	After propensity			After propensity		
	Anakinra (n:120)	Tocilizumab (n: 240)	p	Anakinra (n: 106)	Tocilizumab (n: 106)	p
Hydroxychloroquine, n (%)	81 (67.5)	136 (56.7)	0.048	77 (72.6)	67 (63.2)	0.141
Favipiravir, n (%)	118 (98.3)	240 (100)	0.110	106 (100)	106 (100)	1.000
Glucocorticoid, n (%)	116 (96.7)	195 (81.3)	<0.0001	104 (98.1)	84 (79.2)	<0.0001

## Footnotes

1 Ministry of Health, Turkey. Guidance To Covid-19 (SARS Cov2 Infection) (2021). Website https://hsgm.saglik.gov.tr/tr/covid-19-ingilizce-dokumanlar.html [accessed 27 January 2021]
